# Caregivers’ Perspectives Regarding the Use of Psychotropic Medication in Children and Young Adults: A Systematic Review

**DOI:** 10.1177/00207640251384126

**Published:** 2025-10-31

**Authors:** Phoebe M. Downey, Jack C. Collins, Sarira El-Den, Claire L. O’Reilly

**Affiliations:** 1 The University of Sydney School of Pharmacy, Faculty of Medicine and Health, The University of Sydney, Australia

**Keywords:** psychotropic drugs, child, adolescent, parents, mental health, systematic review

## Abstract

**Background:**

The use of psychotropic medications in children and adolescents is increasing worldwide. However, caregivers’ perspectives regarding the use of these medications in young people remain underexplored.

**Aims:**

To explore caregivers’ perspectives towards the use of psychotropic medications in the treatment of mental disorders in young people.

**Method:**

A systematic search was conducted across five databases (MEDLINE (ALL), Embase, PsycINFO, CINAHL, and Scopus) using a search strategy encompassing the concepts ‘psychotropic medications’, ‘mental health’, ‘attitudes’, ‘young people’, and ‘caregivers’ to identify relevant records published up to 25 March 2024. Records were screened by title and abstract against predefined criteria, followed by full-text review. Data were synthesised and presented in tables. Quality assessment was conducted using the Mixed Methods Appraisal Tool.

**Results:**

After screening 1,296 records, 17 studies were eligible for inclusion. Findings were categorised into caregiver attitudes (*n* = 12), experiences (*n* = 2), and preferences (*n* = 9) regarding the use of psychotropic medications in young people. Most caregivers reported negative attitudes, with scepticism, fears of adverse effects and addiction, and doubts regarding efficacy commonly mentioned, including in those whose children had experience with psychotropic medication. Some caregivers were open to the use of psychotropic medications in their children if it was deemed necessary, however, psychotherapy was overwhelmingly the preferred treatment modality. The methodological quality of included studies was mixed; while many met criteria for appropriate sampling and analysis, few used validated measures, and high risk of bias due to non-response and reporting limitations was common.

**Conclusion:**

This review emphasises the importance of acknowledging and addressing caregiver concerns and actively involving them in the decision-making process. The substantial heterogeneity in study designs, measures, and participant characteristics reinforces the need for future research to employ validated instruments alongside both qualitative and interventional approaches to capture caregiver perspectives and the contextual factors that shape them.

## Introduction

Childhood mental health disorders affect a substantial portion of the global paediatric population, with an estimated prevalence of 13% (Barican et al., 2022; Polanczyk et al., 2015). Reflecting this growing burden, psychotropic medication use in young people has risen globally, however with significant class-specific and cross-national variability (Piovani et al., 2019). In Australia, prescriptions for psychotropic medications in individuals under 18 years have more than doubled between 2013 and 2021 (Wood et al., 2023). Similarly, France reported a 50% rise in monthly psychotropic medication use among 6- to 17-year-olds from 2016 to 2022 (Valtuille et al., 2024). These medications often serve as primary treatments for conditions like attention deficit hyperactivity disorder (ADHD) (May et al., 2023; Wolraich et al., 2019) and conditions involving psychosis (Galletly et al., 2016; Keepers et al., 2020; Malhi et al., 2021), where symptoms cause significant impairment. They are also used alongside psychotherapy for conditions like depression (Hopkins et al., 2015; Malhi et al., 2021) and anxiety (Andrews et al., 2018; Walter et al., 2020) when non-pharmacological treatments alone prove insufficient.

However, the use of psychotropic medications in young people remains contentious, primarily due to limited research on their long-term safety and efficacy, complicated by ethical constraints (Tan & Koelch, 2008). For instance, antidepressants may show reduced effectiveness in adolescents with major depressive disorder compared to adults (Cipriani et al., 2016), and there is an ongoing debate regarding the heightened risk of suicide in this age group (Hetrick et al., 2021). Additional adverse effects include nausea and behavioural activation associated with selective serotonin reuptake inhibitors (SSRIs) (Safer & Zito, 2006), and metabolic disturbances linked to antipsychotics (Bilaç et al., 2023; Morrato et al., 2010). Moreover, medication adherence in paediatric patients is often suboptimal, with reported rates ranging from 5% to 90% (Adler & Nierenberg, 2010; Hamrin et al., 2010; Winnick et al., 2005), although self-reported adherence tends to overestimate actual adherence (Pappadopulos et al., 2009). Factors associated with higher adherence include positive familial attitudes toward treatment and concurrent adherence to psychotherapy (Edgcomb & Zima, 2018).

Despite the need for evidence-based, holistic approaches that integrate psychosocial and pharmacological interventions, many young people still lack adequate support. A recent Australian study found that only 12% of young people with mental health concerns received ‘minimally adequate treatment’, with no significant improvement in quality of life or symptoms of mental illness. Concerningly, only 40% of young people received any treatment at all (Ride et al., 2020). This finding is not in isolation, with poor access to youth mental healthcare remaining a common theme among studies conducted worldwide (Rocha et al., 2015; M. G. Sawyer et al., 2019).

Limited access, coupled with wider cultural and societal factors, can foster caregiver mistrust in the mental healthcare system (Boelsma et al., 2021), particularly among low-income families (Lazar & Davenport, 2018) or ethnic minorities (Jon-Ubabuco & Dimmitt Champion, 2019) who often encounter greater barriers. Sayal et al. (2010) found that many parents felt their concerns were dismissed, leading to a lack of trust and fewer young people receiving necessary care. As caregivers significantly influence their children’s access to treatment (Kalaman et al., 2023), it is essential to understand their perspectives. While patient health beliefs and family support are known predictors of adherence in adult and adolescent populations (Marrero et al., 2020), caregiver perspectives across the critical developmental period from childhood to early adulthood have yet to be systematically synthesised. Accordingly, this systematic review aimed to explore caregivers’ attitudes, experiences, and preferences regarding psychotropic medication use in young people.

## Methods

The systematic review was guided by the Preferred Reporting Items for Systematic Reviews and Meta-Analyses (PRISMA) framework (Page et al., 2021).

### Search Strategy and Eligibility Criteria

The search strategy was developed by one author (PD) in consultation with an academic librarian, then reviewed by members of the research team (JC and COR). PD conducted the search across five databases—MEDLINE (ALL), Embase, and PsycINFO (all via Ovid), CINAHL via EBSCO, and Scopus—on 25 March 2024, with no publication date restrictions. Keywords related to ‘psychotropic medications’, ‘mental health’, ‘attitudes’, ‘young people’, and ‘caregivers’ were combined using Boolean operators. Citation chaining was also employed. Database-specific search strategies are provided as a Supplementary File, in Appendix 1.

Publications meeting the following criteria were included:

(a) Population: caregivers of young people 24 years or younger.
(b) Phenomenon of interest: use of psychotropic medication (actual or hypothetical).
(c) Outcomes: examined perspectives regarding the use of psychotropic medications in young people.
(d) Written in English.
(e) Published in a peer-reviewed journal.

Publications were excluded if they:

(a) Were reviews, non-original research, abstracts, opinion pieces, editorials, non-peer-reviewed theses, or conference presentations.
(b) Focused primarily on psychiatric disorders rather than psychotropic medications.
(c) Examined the views of caregivers of adults.

For this review, ‘children and adolescents’ and ‘young people’ were defined as individuals 24 years or younger, consistent with the extended definition of adolescence encompassing ages 10 to 24 years (S. M. Sawyer et al., 2018). Studies including caregivers of individuals over 24 were excluded unless child and adolescent-specific data were extractable. The term ‘caregiver’ specifically denotes the child’s primary carer, as reported in the included studies.

### Study Selection

Search results were imported into Covidence (Veritas Health Innovation, Melbourne, Australia), and duplicates were removed both automatically and manually. PD screened the remaining records (title/abstract and full text) for inclusion, with 25% of randomly selected excluded records checked by a second reviewer (COR), with no disagreements. Any inclusion queries were resolved through consultation with the research team (JC and COR).

### Data Extraction and Quality Assessment

PD extracted relevant data from eligible studies into tables, including study characteristics (author, year, location, study design, measures, and psychotropic medication focus) and sample characteristics (age, gender, caregiver relationship, education level, and child’s mental health condition). Key findings were summarised in three table outlining caregivers’ attitudes, experiences, and preferences regarding psychotropic medications. The term ‘attitude’ was defined as caregivers’ evaluations of psychotropic medications based on beliefs, emotions, and past behaviours (American Psychological Association, n.d.). These tables were created based on the most frequently explored topics across the included studies, following discussions between two authors (PD and COR).

Quality appraisal was conducted using the Mixed Methods Appraisal Tool (MMAT) 2018 (Hong et al., 2018). PD appraised the included studies, with other authors (JC and COR) reviewing areas of uncertainty. Each item was rated as ‘yes’, ‘no’, or ‘can’t tell’. Following MMAT guidance (Hong et al., 2018), total scores were not calculated; instead, the proportion of studies that met each criterion is presented.

## Results

**Figure 1.**
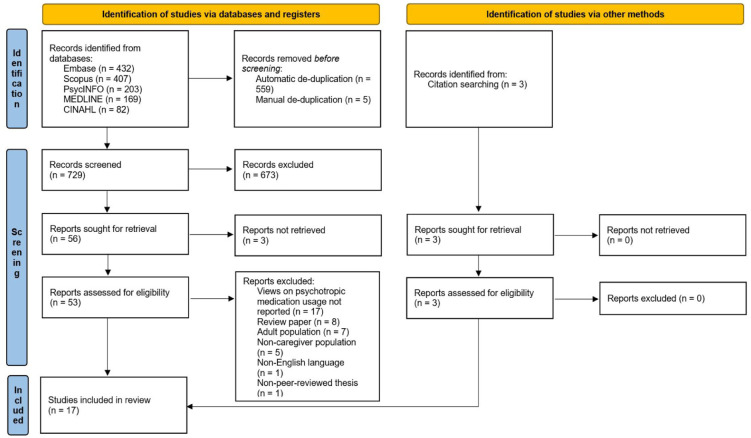
PRISMA 2020 flow diagram for new systematic reviews (Page et al., 2021).

The systematic search identified 1,293 potentially relevant records from Embase (*n* = 432), Scopus (*n* = 407), PsycINFO (*n* = 203), MEDLINE (ALL) (*n* = 169), and CINAHL (*n* = 82), with an additional three records from citation chaining, bringing the total to 1,296. After removing duplicates (*n* = 594), 732 records were eligible for title and abstract screening. A further 673 records were excluded, leaving 59 reports for full-text screening three of which could not be retrieved. Ultimately, 17 studies were eligible for inclusion in this review (Figure 1).

### Study Characteristics

The majority of studies were conducted in the United States (*n* = 10), followed by Australia (*n* = 2) and Saudi Arabia (*n* = 2), and are summarised in Table 1. Most employed cross-sectional designs (*n* = 14), with two longitudinal studies (Chandra et al., 2009; Hilt et al., 2014) and one pre-post interventional study (Talbot & Malas, 2018). Quantitative methods predominated, including written surveys (*n* = 14) and one study using structured telephone interviews (Jorm & Wright, 2007), while two studies used mixed-methods designs incorporating semi-structured interviews alongside surveys (Hilt et al., 2014; Moses, 2011).

**Table 1.** Summarised Study Characteristics of the Included Literature (*n* = 17).

Study (publication year), country	Research type, design	Instrument (no. of items), scale type, instrument validation	Child characteristics *(n, age, gender or sex)*	Mental health conditions under investigation	Caregiver characteristics *(n, age details, gender or sex, relationship to child, education level)*	Hypothetical use or actual use in their child?
Al-Haidar (2008), Saudi Arabia	Quantitative Cross-sectional	Author-developed (8-item) Dichotomous Not validated	NR	NR	*n* = 1,010 30–40 years (51.7%) Gender/sex NR Father (62%) University educated (51%)	Hypothetical use
Al-Harthi et al. (2023), Oman	Quantitative Cross-sectional	Author-developed (8-item) Dichotomous Not validated	*n* = 299 ⩽10 years (44.8%) Male (68.2%)	Neuro-developmental, mood, and psychotic disorders	*n* = 299 30–40 years (52.8% of mothers, 39.8% of fathers) Gender/sex NR Mother (52.2%) Diploma-level education or greater (41.1% of mothers, 41.8% of fathers)	Hypothetical use
A. M. Brown et al. (2007), USA	Quantitative Cross-sectional	TQP-P (adapted from Deacon and Abramowitz (2005)) (items NR) Likert Not validated	*n* = 71 Age range: 5–18 (mean: 12.09, *SD* 3.45) years Male (60.6%)	Anxiety disorders	*n* = 71 Age NR Gender/sex NR Mother (78%) 2-year college degree or greater (91.2% of mothers, 79.4% of fathers)	Actual use
Chandra et al. (2009), USA	Quantitative Longitudinal	Adapted from Jaycox et al. (2006) and Johnson et al. (2006) (9-item) Likert and multiple choice Not validated	*n* = 324 13–15 years (56%) Female (76%)	Varied depression status (major, minor, none)	*n* = 324 Age NR Gender/sex NR All parents High school education or greater (71.6%)	Hypothetical use
Dawood et al. (2020), Saudi Arabia	Quantitative Cross-sectional	Adapted from Lazaratou et al. (2007) (16-item) Dichotomous and Likert Not validated	*n* = 526 Age range: 4–16 (mean: 10.85, *SD* 2.99) years	ADHD, ASD, intellectual disability, learning disability, communication disorder	*n* = 526 Age range: 22–56 (mean: 37.97, *SD*: 9.11) years Female (77.4%) All parents Bachelor’s degree or greater (72.4%)	Actual use
Hilt et al. (2014), USA	Mixed methods Longitudinal	CBCL Parent edition (133-item) and CGI-I (1-item) (both Likert, validated), author-developed qualitative questions (3-item, open response, not validated)	*n* = 255 16–18 years (43.5%) Male (59.9%)	ADHD, anxiety, depression, insomnia, bipolar/mood swings, anger/irritability	*n* = 255 Age NR Gender/sex NR Mother (83.9%) Education NR	Actual use
Jorm and Wright (2007), Australia	Quantitative Cross-sectional	Author-developed (items NR) Likert Not validated	*n* = 1,633 12–17 years^b^ Male (51.1%)	Depression, alcohol misuse, social phobia, psychosis	*n* = 2,005 Age NR Gender/sex NR All parents Education NR	Hypothetical use
Langer et al. (2021), USA	Quantitative Cross-sectional	TPQ-P (adapted from Jaycox et al. (2006); Rapp et al. (2017)) (4-item) Not validated	*n* = 64 Mean age: 11.08 (*SD* 2.23) years Female (54.7%)	Depressive disorders	*n* = 63 Age NR Gender/sex NR Mother (92.1%) Education NR	Actual use
Lazaratou et al. (2007), Greece	Quantitative Cross-sectional	Author-developed (20-item) Likert and multiple choice Not validated	*n* = 134 6–12 years (59.7%) Male (70.1%),	Most commonly reported ICD-10 codes: F80, F81, F93^c^	*n* = 134 25–45 years (79%) Female (83.6%) All parents Highest level of education: high school (40.3%)	Hypothetical use
McLaren et al. (2022), USA	Quantitative Cross-sectional	Author-developed (items NR) Likert Not validated	*n* = 48 6–11 years (50%); 12–17 years (50%) Gender/sex NR	Anxiety or depressive disorders, ADHD, PTSD/stress disorders, ODD, ASD	*n* = 48 40–59 years (56%) Gender/sex NR Relationship to child NR High school education or greater (79%)	Actual use
Moses (2011), USA	Mixed methods^a^ Cross-sectional	Author-developed (17-item) Likert Not validated	*n* = 70 Age range: 12–17 (mean: 14.7, *SD* 1.6) years Male (60%),	ADHD, depression, conduct disorder, bipolar disorder, PTSD, mood disorder NOS, anxiety disorders, ODD, alcohol/drug dependence, reactive attachment disorder	*n* = 70 Age range: 30–70 (mean 43.6, *SD* 9.1) years Gender/sex NR Biological parents (81%) Highest level of education: high school (36%) or some college (36%)	Actual use
O’Brien et al. (2013), USA	Quantitative Cross-sectional	DAI (adapted) (29-item) Likert Not validated	*n* = 18 Mean age: 13.90 (*SD* 3.04) years Male (50%)	Bipolar disorder, depression, ADHD, other	*n* = 19 Mean age: 43.68 (*SD* 9.11) years Female (89.5%) All parents Education NR	Actual use
Post et al. (2002), USA	Quantitative Cross-sectional	Author-developed (items NR) Dichotomous Not validated	N/A	Affective disorders	*n* = 156 Age NR Gender/sex NR All parents Education NR	Hypothetical use
Ricardo Ramírez et al. (2021), Colombia	Quantitative Cross-sectional	Author-developed (25-item) Likert Not validated	NR	Most commonly reported conditions: ADHD, intellectual disability, ODD	*n* = 98 Age range: 19–67 (mean: 39.0, *SD* 10.49) years Female (95%) All parents Highest level of education: high school (51%)	Actual use
Stevens et al. (2009), USA	Quantitative Cross-sectional	Adapted from Nock and Kazdin (2001) (19-item) Likert Not validated	*n* = 501 ⩾11 years (54%) Male (59%)	Primary axis 1 disorders: Internalising, externalising, mixed, other	*n* = 501 Age NR Gender/sex NR High school education or greater (56%) Education NR	Hypothetical use
Talbot and Malas (2018), USA	Quantitative Pre-post intervention	Adapted from Pescosolido et al. (2007) and Turner (2012) (13-item) Likert Not validated	NR	Most frequently reported conditions: anxiety, depression	*n* = 30 45–54 years (50%) Gender/sex NR All parents High-school education or greater (100%)	Hypothetical use
Wallman and Melvin (2022), Australia	Quantitative Cross-sectional	Author-developed (6-item) Likert Not validated	*n* = 143 Age range: 12–18 (mean: 15.51, *SD* 1.74) years Female (55.9%)	NR	*n* = 143 Age range: 32–58 (mean: 44.25, *SD* 6.15) years Female (92.3%) Biological parent (96.5%) Post-graduate degree (34.3%)	Hypothetical use

*Note*. ADHD = Attention Deficit Hyperactivity Disorder; ASD = Autism Spectrum Disorder; CBCL = Child Behaviour Checklist; CGI = Clinical Global Improvement; DAI = Drug Attitude Inventory; ICD = International Classification of Diseases; ITPQ = Initial Treatment Preferences Questionnaire; N/A = Not Applicable; NOS = Not Otherwise Specified; NR = Not Reported; ODD = Oppositional Defiant Disorder; PTSD = Post Traumatic Stress Disorder; TPQ-P = Treatment Perceptions Questionnaire—Parent Version; USA = United States of America.

a Mixed methods design, however only quantitative measures relevant to this review.

b Jorm and Wright (2007) included both adolescents (12–17 years) and young adults (18–25 years). In line with this review’s eligibility criteria, only data for the 12–17-year subgroup were extracted.

c F80 = specific developmental disorders of speech and language, F81 = specific developmental disorders of scholastic skills, F93 = emotional disorders with onset specific to childhood.

Survey instruments varied across studies. Nine studies used non-validated, author-developed surveys, while seven adapted pre-existing instruments—four of which were non-validated and three validated. Likert scales were the most commonly used measurement tool (*n* = 13), assessing caregivers’ views on psychotropic medications, including their efficacy, safety, risks, and treatment preferences.

Ten studies explored the hypothetical use of psychotropic medications. These could be categorised into those examining caregivers of children with diagnosed mental health conditions (Dawood et al., 2020; Lazaratou et al., 2007; Talbot & Malas, 2018), those including caregivers of children both with and without a mental health condition (Al-Haidar, 2008; Al-Harthi et al., 2023; Chandra et al., 2009; Stevens et al., 2009), and those presenting caregivers with hypothetical vignettes without investigating their child’s mental health history (Jorm & Wright, 2007; Post et al., 2002; Wallman & Melvin, 2022). Additionally, seven studies investigated caregiver perspectives on the actual use of psychotropic medications in their own children with mental health conditions.

### Caregiver Characteristics

Caregiver sample sizes varied widely, ranging from 19 to 2005 participants. Sixteen studies reported caregiver demographics, including age, gender or sex, relationship to their child(ren), and educational level. Most caregivers were biological parents, predominantly mothers aged between 30 and 40 years, with the majority having at least a high-school level education.

### Child and Adolescent Characteristics

Child and adolescent sample sizes also varied significantly, ranging from 18 to 1,633 participants. Thirteen studies provided detail on child and adolescent characteristics, with four focusing exclusively on adolescents. Of the 11 studies reporting gender or sex, there was a relatively even distribution between males and females. The most common disorders reported were depressive disorders (*n* = 7), ADHD (*n* = 6), anxiety disorders (*n* = 5), and bipolar disorders (*n* = 3). Mental health conditions were classified using various systems, including International Classification of Diseases Tenth Revision (ICD-10) or broadly categorised as internalising or externalising disorders. A more detailed demographic breakdown is available as a Supplementary File (Appendix 2).

### Caregiver Perspectives

The included studies examined a variety of domains, which were broadly synthesised into: (a) Caregiver attitudes towards the use of psychotropic medications, (b) Caregiver experiences with psychotropic medication treatment, and (c) Caregiver preferences regarding psychotropic medication treatment. Tables 2 to 4 outline the caregiver-centred findings.

**Table 2.** Caregiver Attitudes Surrounding the Use of Psychotropic Medications (*n* = 12).

Study (year), country	Child condition under investigation	Beliefs
Al-Haidar (2008), Saudi Arabia	NR—investigated generalised attitudes towards psychotropic treatment for childhood psychiatric illness.	- Majority believed psychotropic medications should be given to their children if necessary (84.3%). - Reasons for agreeing included trust in psychiatrist knowledge (35.3%), feeling pressure from child’s school (29.5%), belief that psychotropic medications produce faster results than psychotherapy (23.6%), and personal positive experiences with medications (18.1%). - Reasons for objection included concerns about side effects (78.3%), child’s age (71.1%), risk of drug addiction (52%), and belief that mental illness does not occur in children (13.2%).
Al-Harthi et al. (2023), Oman	*Various*: neurodevelopmental disorders, mood disorders, psychotic disorders, and other	- Many believed that psychotropic medications could cause addiction in children (44.1%). - Reported concerns regarding side effects of psychotropic medications (28.4%), including the belief that they cause brain damage (27.4%).
Chandra et al. (2009), USA	Depression	- Fewer Latino and African American parents believed that antidepressants could help treat depression (52%) compared with white parents (90%).
Dawood et al. (2020), Saudi Arabia	*Various*: primarily ADHD, ASD, intellectual disability, learning disability, and communication disorders	- The belief that psychotropic medications are dangerous, causing damage to the brain, stomach, kidneys, and liver (63.7%) and affect the learning abilities of children (64.1%) commonly reported. - Reported belief that psychotropic medications can cause addiction (64.4%), with dependence and abuse being more common in children (79.8%). - Presumed that risks and adverse effects are increased in younger children (90.9%).
Jorm and Wright (2007), Australia	*Four vignettes presented*: depression, depression with alcohol misuse, social phobia, and psychosis.	- Parents had more negative attitudes towards psychotropic medications and more positive views towards SJW compared to their adolescent (12–17-year-old) children. - Antidepressants were viewed most favourably among psychotropic medications. - Many parents of adolescents viewed antidepressants and antipsychotics as harmful for various mental health conditions, particularly in the vignettes of depression, social phobia, and psychosis respectively.
Lazaratou et al. (2007), Greece	*Various*: primarily F80 specific developmental disorders of speech and language, F81 specific developmental disorders of scholastic skills, and F93 emotional disorders with onset specific to childhood	- Parents believed there is excessive use of psychotropic medication (37%) and that doses are unnecessarily high (20%). - Many believed psychotropic medications are dangerous (13% believed all are dangerous; 61% believed specific categories are dangerous). - Believed psychotropic medications can cause addiction: antipsychotics (69%), antidepressants (74%), anxiolytics (57%), hypnotics (82%). - Believed psychotherapy is the most effective treatment for psychological illnesses, including depression (65%), anxiety (68%), and schizophrenia (51%).
Moses (2011), USA	*Various*: ADHD, depression, conduct disorder, bipolar disorder, PTSD, mood disorders, anxiety, ODD, alcohol/drug dependence, and reactive attachment disorder	- Attitudes towards psychotropic medication treatment: mean = 2.3 (*SD* = 0.45) (range:1.4–3.6; lower scores indicate more favourable attitudes).^a^
O’Brien et al. (2013), USA	*Various*: bipolar disorder, depression, ADHD, and other.	- Drug Attitude Inventory mean score slightly above neutral, at 3.95 (*SD* = 0.49) (range: 1–5; higher scores indicate more favourable attitudes).^a^
Post et al. (2002), USA	Affective disorders—vignettes	- Most parents considered psychotropic medications to be required in children at high and very high risk of developing an affective disorder at some stage from the onset of moderate symptoms (44.3%, 61.0%), severe symptoms (65.7%, 81.6%) to definitive diagnosis (92.4% 99.3%).
Ricardo Ramírez et al. (2021), Colombia	*Various*: primarily ADHD, intellectual disability, and ODD.	- Of the parents who believed their child needed some form of treatment, 58.5% believed they needed pharmacotherapy. - Parents whose children had used psychotropic medications believed they may cause adverse effects (64.3%), including addiction (48.7%), ‘slowing down’ (43.2%), ‘change in nature’ (37.8%), worsening of symptoms (21.6%), physical changes (18.9%), sleep problems (8.1%), and irritability (2.7%).
Stevens et al. (2009), USA	*Various primary axis 1 disorders*: internalising disorders, externalising disorder, mixed disorders, and other.	- Described psychotropic medications as both moderately beneficial (mean score = 2.6/4, *SD* = 0.8) and moderately risky (mean score = 2.6/4, *SD* = 0.9). - Belief that psychotropic medications are addictive (68% ‘somewhat’ to ‘strongly’) and can make children want to harm themselves (52% ‘somewhat’ to ‘strongly’) reported.
Talbot and Malas (2018), USA	*Various*: primarily anxiety and depression	- After an educational intervention, there was a significant reduction in stigmatising beliefs towards psychotropic medications: overmedication, from 40% of parents agreeing to 26.7% (*t*(29) = −2.5, *p* = .018), and long term negative effects of psychotropic medication use in youth, from 20% of parents agreeing to 0% (*t*(29) = −2.07, *p* = .048).

*Note*. ADHD = Attention Deficit Hyperactivity Disorder; ASD = Autism Spectrum Disorder; N/A = Not Applicable; NR = Not Reported; ODD = Oppositional Defiant Disorder; SD = Standard Deviation; SJW = St John’s Wort; USA = United States of America.

a Scale for these measures is not available.

**Table 3.** Caregiver Experiences With Psychotropic Medications (*n* = 2).

Study (year), country	Child condition	Experiences
Hilt et al. (2014), USA	*Various*: ADHD, anxiety, depression, insomnia, bipolar/mood swings, and anger/irritability.	- Majority of caregivers noted symptom improvement in their children with psychotropic medication, while majority of children demonstrated at least one CBCL scale score in the clinically signiﬁcant range, indicating that psychotropic polypharmacy may not be adequately providing symptom improvement. - 47% of medications were producing unwanted side effects, majority of which had been used for ⩾6 months.
McLaren et al. (2022), USA	*Various*: anxiety or depressive disorders, ADHD, PTSD/stress disorders, ODD, and ASD	- Concerns regarding the efficacy (44%), side effects (38%), and long-term consequences (35%) of their child’s psychotropic medication use, including polypharmacy (31%) reported. - Many caregivers had their child stop taking their psychotropic medication(s) early (42%), without the prescriber’s guidance (due to lack of efficacy (50%), adverse effects (50%), and child’s refusal to take (35%)).

*Note*. ADHD = Attention Deficit Hyperactivity Disorder; ASD = Autism Spectrum Disorder; CBCL = Child Behaviour Checklist; ODD = Oppositional Defiant Disorder; PTSD = Post Traumatic Stress Disorder; USA = United States of America.

**Table 4.** Caregiver Preferences Regarding Psychotropic Medications (*n* = 9).

Study (year), country	Child condition	Preferences
Al-Haidar (2008), Saudi Arabia	NR—investigated generalised attitudes towards psychotropic treatment for childhood psychiatric illness.	- Psychotherapy preferred initial treatment (83.5%). - Many parents preferred consult with folk healers over psychiatrists (25.7%).
Al-Harthi et al. (2023), Oman	*Various*: neurodevelopmental disorders, mood disorders, psychotic disorders, and other	- Psychotherapy preferred as initial treatment (91.6%). - Some parents reported desire to consult folk healer before a psychiatrist (24.1%).
Brown et al. (2007), USA	Various anxiety disorders	- Caregivers preferred CBT alone (54.5%) over pharmacotherapy alone (7.3%) and combination (38.2%) as initial treatment. - Rated CBT as more acceptable than psychotropic medication.
Chandra et al. (2009), USA	Depression	- Latino and African American parents preferred watchful waiting (22%, 24%) or counselling (66%, 62%) whereas White parents preferred combination counselling and antidepressant treatment (45%) or counselling alone (45%).
Langer et al. (2021), USA	Depression	- Preferred psychotherapy (76.2%) and combined psychotherapy alongside medication (19%) over medication alone (0%). - Higher perceived symptom severity associated with greater likelihood of preferring combined treatment.
Post et al. (2002), USA	Affective disorders	- Preferred acute counselling at onset of moderate symptoms of an affective disorder, long-term counselling and acute psychotropic medication use at onset of moderate-severe symptoms, and long-term psychotropic medication use at onset of severe symptoms. - SSRIs, bupropion, lithium, carbamazepine, and valproate were preferred for high-risk children at onset of severe symptoms. TCAs were preferred for children between the onset of severe symptoms and at diagnosis, while lamotrigine, gabapentin, and CCBs were preferred only at diagnosis.
Ricardo Ramírez et al. (2021), Colombia	*Various*: primarily ADHD, intellectual disability, and ODD.	- Psychotherapy first preference (90.4%), then pharmacotherapy (58.5%). - ‘Healing’ (traditional spiritual practices) was the preferred alternative treatment (27.5%), followed by dietary changes (24.5%).
Stevens et al. (2009), USA	*Various primary axis 1 disorders*: internalising disorders, externalising disorder, mixed disorders, and other.	- Preferred antidepressants over counselling (40%); counselling over antidepressants (24%). - Those with a child who was depressed or taking an antidepressant reported higher benefits for counselling and reduced perceived risks.
Wallman and Melvin (2022), Australia	Depression	- Preferred exercise first, then counselling across all depression vignettes. - Pharmacotherapy was consistently the third treatment preference, with preference increasing alongside depression severity (mean preference ratings: 2.90, 6.62, 6.62). - Antidepressants were preferred for severe and treatment-resistant depression (rated 6.62/10) over mild and moderate depression (rated 2.98/10). - Antidepressants were more strongly preferred by those with previous positive experiences with pharmacotherapy (effectiveness, fewer adverse effects).

*Note*. ADHD = Attention Deficit Hyperactivity Disorder; CBT = Cognitive Behavioural Therapy; CCB = Calcium Channel Blocker; N/A = Not Recorded; NR = Not Reported; ODD = Oppositional Defiant Disorder; PTSD = Post Traumatic Stress Disorder; SSRI = Selective Serotonin Reuptake Inhibitor; TCA = Tricyclic Antidepressant; USA = United States of America.

### Caregiver Attitudes Towards Psychotropic Medication Treatment

Twelve studies examined caregiver attitudes towards psychotropic medication use, with many caregivers expressing scepticism, particularly regarding the perceived risks of these medications (Table 2). Concerns regarding the addictive potential of psychotropic medications were common, reported in six studies. These concerns were not limited to medications with known addictive properties; for example, Lazaratou et al. (2007) found that parents commonly believed antidepressants and antipsychotics to be addictive, a sentiment echoed by Stevens et al. (2009) regarding antidepressants. Fears of severe and long-term adverse effects were also prevalent, as noted in six studies. Caregivers worried about potential multisystem damage, including brain and liver damage (Al-Harthi et al., 2023; Dawood et al., 2020), as well as generalised dysfunction (Ricardo Ramírez et al., 2021). Some caregivers worried that psychotropic medications could exacerbate mental illness or lead to personality changes (Ricardo Ramírez et al., 2021; Stevens et al., 2009). Parental distrust of psychiatrists emerged in two studies, with caregivers believing that psychiatrists tend to overprescribe medications at unnecessarily high doses (Dawood et al., 2020; Lazaratou et al., 2007). Additionally, Jorm and Wright (2007) observed that parents held more negative views toward psychotropic medications compared to their adolescent children, particularly viewing them as harmful across various mental health conditions.

Conversely, some studies indicated more favourable attitudes among caregivers. Moses (2011) and M. O’Brien et al. (2013) reported generally positive or neutral attitudes toward psychotropic medications. Moreover, in two studies, caregivers acknowledged the necessity of psychotropic medications under specific circumstances. Post et al. (2002) reported that parents supported psychotropic pharmacotherapy for hypothetical children with a high (20%–30%) or very high (70%) risk of developing an affective disorder, depending on disease progression. Similarly, Al-Haidar (2008) found that parents often sought psychotropic medications to comply with authority figures, such as psychiatrists or the education system. The desire for faster symptom improvement than they believed psychotherapy could provide, along with positive personal experiences with psychotropic medications also influenced their attitudes.

The relationship between caregiver educational attainment and attitudes was explored in six studies, yielding mixed results. While Al-Harthi et al. (2023) found a significant association between lower educational attainment and negative attitudes, Lazaratou et al. (2007) contrastingly found a significant association with higher educational attainment. Ultimately, the majority of studies that investigated this relationship found no statistically significant relationship.

Only one interventional study, conducted by Talbot and Malas (2018), was included in this review. After a brief educational video, a significant reduction in stigmatising attitudes and beliefs towards psychotropic medications was noted, particularly in relation to beliefs regarding psychotropic overmedication and the risk of long-term adverse effects, overall increasing parental confidence in paediatric psychiatry.

### Caregiver Experiences With Psychotropic Medication Treatment

Two studies investigated caregivers’ firsthand experiences with psychotropic medications for their children (Table 3). McLaren et al. (2022) reported that while many caregivers felt informed about the medications’ mechanisms and purposes, nearly half (44%) doubted their efficacy. In some instances (42%), caregivers discontinued their child’s medication without consulting healthcare providers, primarily due to perceived ineffectiveness, adverse effects, or the child’s refusal to continue treatment. Hilt et al. (2014) found that while caregivers often reported symptom improvement in their children using the Clinical Global Improvement scale, persistent mood and behavioural concerns were evident when assessed with the Child Behaviour Checklist over a 15-month period. This discrepancy indicates that while short-term improvements are perceived, long-term or underlying issues may not be fully addressed by medication alone.

### Caregiver Preferences Regarding Psychotropic Medication Treatment

Nine studies investigated caregiver preferences between treatment modalities, with the majority comparing non-pharmacological and pharmacological treatments (Table 4). A strong preference for psychotherapy over pharmacotherapy emerged, both as an initial and general treatment option. For instance, Al-Haidar (2008) reported that 83.5% of parents preferred psychotherapy as a first-line treatment, and Al-Harthi et al. (2023) found a similar preference rate of 91.6%. Post et al. (2002) observed that on average, parents preferred acute counselling at the onset of moderate affective symptoms and long-term counselling at the onset of moderate to severe symptoms. Preferences shifted with increasing disease severity; Wallman and Melvin (2022) found that caregivers were more open to pharmacotherapy for more severe conditions, and Langer et al. (2021) noted an association between preference for combined therapy and higher symptom severity.

The preference for folk healer consultation was observed in three studies, with two investigating this as a preference to see a folk healer over a psychiatrist (Al-Haidar, 2008; Al-Harthi et al., 2023) and one describing it as the preferred form of alternative treatment (Ricardo Ramírez et al., 2021). It should be noted, however, that despite the preference for treatment through a folk healer, Al-Haidar (2008) found that caregivers were regardless more likely to give their children psychotropic pharmacotherapy if it was perceived as necessary. Preference for folk healer consultation was associated with a lower education level in the analysis conducted by Al-Harthi et al. (2023).

Some caregivers’ personal experiences with psychotropic medications influenced their preferences. Those with positive experiences were more likely to view pharmacotherapy favourable (Al-Haidar, 2008; Stevens et al., 2009; Wallman & Melvin, 2022), suggesting that firsthand experience can mitigate some concerns and increase acceptance of medication as a viable treatment option.

### Quality Assessment

Among the 14 quantitative descriptive studies, ten used sampling strategies that were relevant to the research question (Q1) and achieved samples representative of their target populations (Q2), and all applied appropriate statistical methods (Q5). However, only five employed validated instruments to measure caregiver attitudes, experiences, or preferences (Q3). Likewise, only five studies were considered to have a low risk of non-response bias (Q4).

The single quantitative non‑randomised study satisfied the criteria for sampling representativeness (Q1), use of appropriate measurements (Q2), and its intervention was delivered as planned (Q5), however only two-thirds of respondents provided complete outcome data (Q3), and the study report did not specify whether or how confounders were controlled (Q4).

Both mixed-methods studies lacked a clear rationale for using a mixed methods design (Q1), failed to produce integrated analyses (Q2−Q3), and had methodological flaws in individual study components (Q5). However, both studies adequately addressed inconsistencies between qualitative and quantitative findings (Q4–Q5). Full item‑level MMAT ratings are available in Supplementary File (Appendix 3).

## Discussion

As the use of psychotropic medications for young people remains a topic of significant debate, understanding the perspectives of those most involved in their care is crucial. This review explores caregivers’ attitudes, experiences, and preferences regarding these medications. While some caregivers believed in their utility in certain circumstances, the prevailing sentiment leaned towards scepticism and apprehension, with many expressing concerns regarding potential risks.

A primary concern identified among caregivers pertained to the potential adverse effects of psychotropic medications in young people. Specifically, many caregivers expressed apprehension regarding long-term consequences, such as potential brain damage, despite the current paucity of research on their impact on the developing brain. While some evidence indicates that antipsychotics may be associated with reduced brain volume in adults with schizophrenia (Ho et al., 2011; Moncrieff & Leo, 2010), the implications for young people remain unclear. Additionally, caregivers expressed concerns regarding the risk of negative personality changes with psychotropic medications, however evidence suggests that both psychotherapy and pharmacotherapy may positively affect personality traits in adults (Roberts et al., 2017), although similar effects in young people are not well established. Caregivers also raised concerns about the risk of worsening mental illness symptoms. This aligns with concerns regarding the association between antidepressant use and suicidality in young people (Sharma et al., 2016). However, the extent of this risk, whether it applies to all antidepressants (Boaden et al., 2020), and the impact of other confounding factors remains unclear, as many studies exclude individuals at risk of suicide (Hetrick et al., 2021). Compounding these concerns, caregivers’ exposure to potential misinformation online may also distort their understanding of psychotropic medication risks and benefits, potentially heightening undue apprehension and steering choices away from evidence-based recommendations (Frey et al., 2022; Wang et al., 2019). Given these multifactorial concerns, practitioners should carefully weigh the potential risks and benefits of psychotropic medications, taking into account the unique needs and circumstances of each young patient.

The fear of addiction to psychotropic medications was frequently raised among caregivers. It is important to differentiate between addiction, which involves uncontrollable drug use despite negative consequences, and dependence, marked by withdrawal symptoms upon drug cessation (C. P. O’Brien et al., 2006). Certain psychotropic medications, such as SSRIs and antipsychotics, can cause physical dependence, necessitating a gradual dose reduction to avoid withdrawal symptoms (Cosci & Chouinard, 2020). However, true addiction risk is primarily associated with specific classes of medications like benzodiazepines (Blanco et al., 2018) and psychostimulants (Compton et al., 2018). Notably, despite the addiction risk associated with psychostimulants, early use of these medications for ADHD may reduce the risk of future substance use disorders (Chang et al., 2014; Faraone & Wilens, 2007; Wilens et al., 2003), with misuse typically linked to pre-existing conduct disorders (Roy, 2008). Educating caregivers about these nuances is essential to address misconceptions, particularly regarding antidepressant and antipsychotic medications. In one of the included studies, caregivers believed that antidepressants and antipsychotics had the highest addiction risk (Lazaratou et al., 2007), highlighting a significant gap in understanding. By providing clear and accurate information about the actual risks associated with different psychotropic medications, healthcare providers can alleviate unfounded fears and promote adherence to treatment plans. Addressing these misconceptions is vital to ensure that children and adolescents receive timely and appropriate interventions without unnecessary delays or discontinuations due to caregiver concerns.

Furthermore, some caregivers expressed doubts regarding the efficacy of psychotropic medications, with McLaren et al. (2022) reporting that these doubts led some to discontinue their child’s medication without professional guidance. While a recent review of various psychotropic medications showed efficacy for the treatment of some mental illnesses in young people, particularly for the use of psychostimulants in ADHD, much of the evidence was of low quality with small effect sizes (Correll et al., 2021). A recent network meta-analysis conducted by Hetrick et al. (2021) suggests that newer antidepressants may have a slight effect in reducing depressive symptoms in young people, with little variation between classes. Similarly, second-generation antipsychotics have shown acute efficacy for psychosis, mania, and aggression in young people, but data on long-term efficacy remain scarce (Singappuli et al., 2022; Vitiello et al., 2009). Given this uncertainty, transparent conversations between healthcare professionals and caregivers are critical to ensure informed decision-making.

Many caregivers expressed a strong preference for non-pharmacological treatments, such as psychotherapy, particularly for conditions like mild to moderate depression and anxiety, in line with clinical guidelines (Andrews et al., 2018; Hopkins et al., 2015; Malhi et al., 2021; Walter et al., 2020). While acknowledging the role of psychotropic medications in certain cases, the majority preferred these alternatives, especially as first-line treatments. However, several barriers complicate access to non-pharmacological care, including long waiting periods, financial constraints, lack of awareness, shame, distrust of the service system, and poor communication between services and families (A. Brown et al., 2016; Hansen et al., 2021; Iskra et al., 2018; Savaglio et al., 2023). These obstacles not only delay the initiation of appropriate treatment (Westberg et al., 2022) but also undermine the effectiveness of early intervention, which is crucial for optimal long-term outcomes (Colizzi et al., 2020; Fusar-Poli, 2019). Addressing these barriers is essential for improving access to care and ensuring that young people receive timely, effective treatment.

Some caregivers expressed a preference for consultations with traditional folk healers over psychiatrists, particularly in studies conducted in the Middle East (Oman and Saudi Arabia). A recent systematic review conducted by Elshamy et al. (2023) found that many individuals of Middle Eastern cultures first seek help for mental health concerns from alternative healers, such as traditional and religious healers, prior to consulting a healthcare professional. The understanding of mental illness itself is often rooted in long-standing religious and cultural contexts, making these alternative methods of treatment more culturally acceptable as well as more financially and geographically accessible. Similarly, one of the studies included in this review, conducted in Colombia by Ricardo Ramírez et al. (2021) found that ‘healing’ was the preferred adjuvant treatment among their sample of parents, referring to traditional spiritual healing practices. Similar barriers to accessing mental health care in the Middle East are also noted in many countries in Latin America, including Colombia, with factors such as stigma and affordability playing a role (Campo-Arias et al., 2020). Traditional healers tend to be able to provide more culturally and linguistically compatible care (Cruz et al., 2022), and hence, healthcare providers should be aware of the structural barriers associated with the biomedical model of healthcare (Carbonell et al., 2020) and work towards addressing systemic structural issues, allowing for improved access.

It is crucial to recognise that caregiver attitudes towards psychotropic medications can shape patterns of adherence, with more favourable views associated with greater medication persistence and reduced risk of early discontinuation (Kalaman et al., 2023). Given the complex interplay of factors affecting adherence, practitioners must first address caregiver beliefs while considering the broader family context (Charach et al., 2008). Shared decision-making serves as one key avenue for this, fostering a collaborative environment where families can explore treatment options, weigh benefits and harms, discuss their personal values, and make informed decisions alongside clinicians (Langer & Jensen-Doss, 2018; Liverpool et al., 2021; Yoo et al., 2018). Yet while widely endorsed, shared decision-making remains inconsistently implemented (Goossensen et al., 2007), presenting an opportunity for improvement, particularly through the use of structured frameworks such as the NICE Shared Decision Making guideline (Carmona et al., 2021). Although interventional studies remain sparse, early findings are encouraging; the sole interventional study included in this review reported a significant reduction in stigmatising attitudes following a caregiver-focused psychoeducation session (Talbot & Malas, 2018), albeit with limitations in design and generalisability. More established condition-specific programmes, such as those targeting ADHD, have demonstrated positive impacts on both adherence and symptom management (Bai et al., 2015), illustrating how engaging caregivers meaningfully can translate into tangible therapeutic gains. Collectively, these findings highlight the need to embed structured, values-based strategies such as shared decision-making and psychoeducation into routine care, supporting caregiver engagement as a means of promoting informed treatment choices and sustained adherence.

It should be noted that many studies included in this review relied on non-validated, closed-ended, self-reported survey instruments and were predominantly cross-sectional. These methodological choices may have constrained participant responses, limited depth of insight, and introduced social-desirability and recall biases (Althubaiti, 2016; Panacek, 2008). To gain a deeper understanding of caregiver attitudes, further high-quality research using validated instruments and qualitative methods is necessary.

### Strengths and Limitations

Despite several strengths, including the transparency and reproducibility afforded by following the PRISMA guidelines (Page et al., 2021), this review is not without limitations. While the included studies were diverse in their settings, most were conducted in Western countries, as only English-language studies were included, limiting generalisability. Additionally, only one author was responsible for screening and data extraction, although uncertainties were resolved in consultation with three authors. Nonetheless, this systematic review presents a comprehensive understanding of caregivers’ attitudes towards, experiences with, and preferences regarding the use of psychotropic medications in young people.

## Conclusion

This systematic review highlights the importance of involving caregivers in the decision-making process surrounding the use of psychotropic medications in their children. Caregivers often expressed negative sentiments towards these medications, citing concerns about adverse effects, risk of addiction, efficacy, and overprescribing. These concerns, at times, led to early cessation without healthcare provider guidance, illustrating the importance of ongoing support. Embedding shared decision-making and culturally responsive psychoeducation into routine care may help address caregiver concerns and promote more collaborative, sustained use of medication. Further high-quality research using validated tools and qualitative methods is needed to gain a more comprehensive understanding of caregivers’ attitudes towards psychotropic medications and the degree to which these strategies can modulate perspectives in practice.

## Supplemental Material

sj-docx-1-isp-10.1177_00207640251384126 – Supplemental material for Caregivers’ Perspectives Regarding the Use of Psychotropic Medication in Children and Young Adults: A Systematic Review Supplemental material, sj-docx-1-isp-10.1177_00207640251384126 for Caregivers’ Perspectives Regarding the Use of Psychotropic Medication in Children and Young Adults: A Systematic Review by Phoebe M. Downey, Jack C. Collins, Sarira El-Den and Claire L. O’Reilly in International Journal of Social Psychiatry
