# Supplementary material for: Caregivers’ Perspectives Regarding the Use of Psychotropic Medication in Children and Young Adults: A Systematic Review
Source: Int J Soc Psychiatry. 2025 Oct 31;72(4):728–44. doi: 10.1177/00207640251384126 (PMC13263459; doi:10.1177/00207640251384126)
Supplement: sj-docx-1-isp-10.1177_00207640251384126 – Supplemental material for Caregivers’ Perspectives Regarding the Use of Psychotropic Medication in Children and Young Adults: A Systematic Review [file sj-docx-1-isp-10.1177_00207640251384126.docx]

**Appendix 1**

Overview of search strategies used for each database (MEDLINE (ALL), Embase, and PsycINFO hosted by Ovid, CINAHL vis EBSCO, and Scopus conducted on March 25, 2024.

| **Database** | **Search Strategy** |
| --- | --- |
| **MEDLINE (ALL) hosted by Ovid** | **1**  (youth or "young adult*" or adolescent* or p?ediatric* or child or children or teen*).mp. (4524426)  **2**  (parent* or mother* or father* or guardian* or caregiver* or carer* or grandparent* or "foster parent*" or "informal caregiver*").mp. (936449)  **3**  psychotropic drugs/ or antidepressive agents/ or anti-anxiety agents/ or antipsychotic agents/ (142902)  **4**  (antidepressant* or anxiolytic* or "psychotropic drug*" or "psychotropic medic*" or "psychoactive medic*" or antipsychotic* or "mood stabili?er*" or psychopharmaceutical* or "psychoactive drug*").mp. (190903)  **5**  3 or 4 (217040)  **6**  ("mental health" or "mental illness*" or "mental disorder*" or depression or anxiety or schizophrenia or schizoaffective or "bipolar disorder").mp. (1195210)  **7**  (attitude* or opinion* or belief* or believe* or view* or perspective*).mp. (1434590)  **8**  1 and 2 and 5 and 6 and 7 (169) |
| **Embase hosted by Ovid** | **1**  (youth or "young adult*" or adolescent* or p?ediatric* or child or children or teen*).mp. (5036341)  **2**  (parent* or mother* or father* or guardian* or caregiver* or carer* or grandparent* or "foster parent*" or "informal caregiver*").mp. (1298996)  **3**  psychotropic agent/ or antidepressant agent/ or anxiolytic agent/ or neuroleptic agents (235236)  **4** (antidepressant* or anxiolytic* or "psychotropic drug*" or "psychotropic medic*" or "psychoactive medic*" or antipsychotic* or "mood stabili?er*" or psychopharmaceutical* or "psychoactive drug*").mp. (310243)  **5** 3 or 4 (358713) **6** ("mental health" or "mental illness*" or "mental disorder*" or depression or anxiety or schizophrenia or schizoaffective or "bipolar disorder").mp. (1764370)  **7**(attitude* or opinion* or belief* or believe* or view* or perspective*).mp. (2051033)  **8**1 and 2 and 5 and 6 and 7 (432) |
| **PsycINFO (hosted by Ovid)** | **1**  (youth or "young adult*" or adolescent* or p?ediatric* or child or children or teen*).mp. (1031698)  **2**  (parent* or mother* or father* or guardian* or caregiver* or carer* or grandparent* or "foster parent*" or "informal caregiver*").mp. (513240)  **3**  psychotropic drugs/ or antidepressant drugs/ or anxiolytic drugs/ or neuroleptic drugs/ (49413)  **4**  (antidepressant* or anxiolytic* or "Psychotropic Drug*" or "psychotropic medic*" or "psychoactive medic*" or antipsychotic* or "mood stabili?er*" or psychopharmaceutical* or "psychoactive drug*").mp. (111466)  **5**  3 or 4 (115639)  **6**  ("mental health" or "mental illness*" or "mental disorder*" or depression or anxiety or schizophrenia or schizoaffective or "bipolar disorder").mp. (1065607)  **7**  (attitude* or opinion* or belief* or believe* or view* or perspective*).mp. (1434590)  **8**  1 and 2 and 5 and 6 and 7 (203) |
| **CINAHL via EBSCO** | ((youth OR "young adult*" OR adolescent* OR p#ediatric* OR child OR children OR teen*)) AND ((parent* OR mother* OR father* OR guardian* OR caregiver* OR carer* OR grandparent* OR "foster parent*" OR "informal caregiver*")) AND ((MH "psychotropic drugs") OR (MH "antidepressant drugs") OR (MH "anxiolytic drugs") OR (MH "neuroleptic drugs") OR (antidepressant* OR anxiolytic* OR "psychotropic drug*" OR "psychotropic medic*" OR "psychoactive medic*" OR antipsychotic* OR "mood stabili#er*" OR psychopharmaceutical* OR "psychoactive drug*")) AND (("mental health" OR "mental illness*" OR "mental disorder*" OR depression OR anxiety OR schizophrenia OR schizoaffective OR "bipolar disorder")) AND ((attitude* OR opinion* OR belief* OR believe* OR view* OR perspective*)) (82) |
| **Scopus** | ( youth OR "young adult*" OR adolescent* OR p?ediatric* OR child OR children OR teen* ) AND ( parent* OR mother* OR father* OR guardian* OR caregiver* OR carer* OR grandparent* OR "foster parent*" OR "informal caregiver*" ) AND ( antidepressant* OR anxiolytic* OR “psychotropic” OR "psychotropic drug*" OR "psychotropic medic*" OR "psychoactive medic*" OR antipsychotic* OR "mood stabili?er*" OR psychopharmaceutical* OR "psychoactive drug*" ) AND ( "mental health" OR "mental illness*" OR "mental disorder*" OR depression OR anxiety OR schizophrenia OR schizoaffective OR "bipolar disorder" ) AND ( attitude* OR opinion* OR belief* OR believe* OR view* OR perspective* ) (407) |

**Appendix 2**

**Table 1:** Study characteristics of the included literature (n=17)

| **Study (publication year), country** | **Research type, design** | **Instrument (no. of items), scale type, instrument validation** | **Child characteristics *(n, age, gender or sex)*** | **Child mental health condition** | **Caregiver characteristics *(n, age, gender or sex, relationship to child, education level)*** | **Hypothetical use or actual use in their child?** |
| --- | --- | --- | --- | --- | --- | --- |
| **Al-Haidar (2008),** Saudi Arabia | Quantitative  Cross-sectional | Author-developed (8-item)  Dichotomous  Not validated | NR | NR | **n=**1010  **Age:**  - <30 years: 227 (23.3%)  - 30-40 years: 503 (51.7%)  - >40-50 years: 190 (19.5%)  - >50 years: 53 (5.4%)  **Gender**: NR  **Relationship to child:**  - Mother: 369 (38.0%)  - Father: 603 (62.0%)  **Education:**  - Illiterate: 30 (3.0%)  - Elementary: 50 (5.0%)  - Secondary: 132 (13.3%)  - High school: 271 (27.2%)  - University: 433 (43.5%)  - Higher education: 79 (7.9%) | Hypothetical use |
| **Al-Harthi et al. (2023),** Oman | Quantitative  Cross-sectional | Author-developed (8-item)  Dichotomous  Not validated | **n=**299  **Age:**  - ≤ 10 years: 134 (44.8%)  - 11-15 years: 94 (31.4%)  - 16-18 years: 71 (23.7%)  **Gender**: Male (204, 68.2%), female (95, 31.6%) | Neuro-developmental disorders, mood disorders, psychotic disorder, other (e.g., epilepsy, genetic syndromes) | **n=**299  **Age:**  Father:  - < 30 years: 7 (2.3%)  - 30-40 years: 119 (39.8%)  - 41-50 years: 119 (39.8%)  - > 50 years: 54 (18.1%)  Mother:  - < 30 years: 20 (6.7%)  - 30-40 years: 158 (52.8%)  - 41-50 years: 113 (37.8%)  - > 50 years: 54 (2.7%)  **Gender:** NR  **Relationship to child:**  - Father: 117 (39.1%)  - Mother: 156 (52.2%)  - Other caregiver: 26 (8.7%)  **Education:**  Father: illiterate-grade 11: 76 (25.4%), grade 12: 98 (32.8%): diploma or higher 125 (41.8%)  Mother: illiterate-grade 11: 71 (23.7%), grade 12: 105 (35.1%), diploma or higher: 123 (41.1%) | Hypothetical use |
| **Brown et al. (2007),** USA | Quantitative  Cross-sectional | TQP-P (adapted from Deacon and Abramowitz (2005)) (items NR)  Likert  Not validated | **n=**71  **Age range:** 5-18 (mean: 12.09, SD 3.45) years  **Gender:** Male (43, 60.6%), female (28, 39.4%) | **Anxiety disorders:**  OCD, GAD, separation anxiety disorder, social phobia, specific phobia, panic disorder, other | **n=**71  **Age**: NR  **Gender:** NR  **Relationship to child:** NR  - Mother: 78%  **Education:**  - Mother: 2-year college degree or higher:  - Father: 2-year college degree or higher: 91.2% mothers, 79.4% fathers | Actual use |
| **Chandra et al. (2009),** USA | Quantitative  Longitudinal | Adapted from Jaycox et al. (2006) and Johnson et al. (2006) (9-item)  Likert and multiple choice  Not validated | **n=**324  **Age:**  - 13-15 years: 182 (56%)  - 16-18 years: 142 (43.8%)  **Gender:** Male (78, 24%), female (246, 76%) | **Depression status:** not depressed, minor depression, major depression | **n=**324  **Age:** NR  **Gender:** NR  **Relationship to child**: all parents  **Education:**  - Did not finish high school: 92 (28.4%)  - High school graduate/GED: 86 (26.5%)  - Some college/AA degree: 76 (23.4%)  - 4-year college degree: 39 (12.0%)  - Graduate/professional degree: 31 (9.5%) | Hypothetical use |
| **Dawood et al. (2020),** Saudi Arabia | Quantitative  Cross-sectional | Adapted from Lazaratou et al. (2007) (16-item)  Dichotomous and Likert  Not validated | **n=**526  **Age range**: 4-16 (mean: 10.85, SD 2.99) years  **Gender:** NR | ADHD, ASD, intellectual disability, learning disability, communication disorder | **n=**526  **Age range**: 22-56 years (mean=38, SD=9.11 years)  **Gender:** Male (119, 22.6%), female (407, 77.4%)  **Relationship to child**: all parents  **Education**: illiterate (n=10), diploma (n=135), bachelor’s degree (n=361), master’s degree (n=16), PhD (n=4) | Actual use |
| **Hilt et al. (2014),** USA | Mixed methods  Longitudinal | CBCL Parent edition (133-item) and CGI-I (1-item) (both Likert, validated), author-developed qualitative questions (3-item, open response, not validated) | **n(t_0_)**=255  **Age range (t_0_):**  - 6-10 years: 19.8%  - 11-15 years: 36.6%  - 16-18 years: 43.5%)  **Sex (t_0_)**: Male (59.9%), female (40.1%) | ADHD, anxiety, depression, insomnia, bipolar/mood swings: anger/irritability | **n (t_0_)** = 255  **Age:** NR  **Sex:** NR  **Relationship to child (t_0_):**  - Mother = 83.9%  - Father = 14.5%  - Other legal guardian=1.6%  **Education**: NR | Actual use |
| **Jorm and Wright (2007),** Australia | Quantitative  Cross-sectional | Author-developed (items NR)  Likert  Not validated | **n=**1633  **Age range:** 12-17 years  **Gender:** Male (835, 51.1%), female (798, 48.9%) | N/A | **n=**2005  **Age**: NR  **Gender:** NR  **Relationship to child:** all parents  **Education**: NR | Hypothetical use |
| **Langer et al. (2021),** USA | Quantitative  Cross-sectional | TPQ-P (adapted from Rapp et al. (2017); Jaycox et al. (2006))  (4-item)  Not validated | **n**= 64  **Mean age:** 11.08 (SD 2.23) years  **Gender:** Male (29, 45.3%), female (35, 54.7%) | **Depressive disorders:**  MDD, Dysthymic Disorder, Depressive Disorder NOS | **n**=63  **Age**: NR  **Gender:** NR  **Relationship to child**: 92.1% mothers, 6.3% fathers, 1.5% grandparent  **Education:** NR | Actual use |
| **Lazaratou et al. (2007),** Greece | Quantitative  Cross-sectional | Author-developed (20-item)  Likert and multiple choice  Not validated | **n**=134  **Age**:  - ≤ 5 years: 26 (19.4%)  - 6-12 years: 80 (59.7%)  - 12-18 years: 28 (20.9%)  **Sex:** Male (94, 70.1%), female (40, 29.9%) | **Most commonly reported conditions (ICD-10 classification system):**  F80 specific developmental disorders of speech and language, F81 specific developmental disorders of scholastic skills, F93 emotional disorders with onset specific to childhood | **n**=134  **Age:**  - < 25 years: 8 (6%)  - 25-45 years: 106 (79%)  - > 45 years: 20 (15%)  **Sex:** Male (22, 16.4%), female (112, 83.6%)  **Relationship to child**: all parents  **Education**:  - Elementary = 41 (30.6%)  - High school = 54 (40.3%)  - University (29.0%) | Hypothetical use |
| **McLaren et al. (2022),** USA | Quantitative  Cross-sectional | Author-developed (items NR)  Likert  Not validated | **n=**48  **Age:**  - 6-11 years: 50%  - 12-17 years: 50%  **Gender**: NR | Anxiety or depressive disorders, ADHD, PTSD/stress disorders, ODD, ASD | **n=**48  **Age**  - 40-59 years: 56%  **Gender**: NR  **Education:** greater than high school: 79% | Actual use |
| **Moses (2011),** USA | Mixed methods ^a^  Cross-sectional | Author-developed (17-item)  Likert  Not validated | **n**=70  **Age range**: 12-17 (mean: 14.7, SD 1.6) years  **Gender**: Male (40, 60%), female (30, 40%) | ADHD, depression, conduct disorder, bipolar disorder, PTS, mood disorder NOS, anxiety disorders, ODD, alcohol/drug dependence, reactive attachment disorder | **n**=70  **Age range:** 30-70 (mean 43.6, SD 9.1) years  **Gender:** NR  **Relationship to child:**  - Biological parent: 57 (81%)  - Adoptive parent: 6 (9%)  - Kin: 7 (10%)  **Education:**  - High school (or equivalent): 25 (36%)  - Some college: 25 (36%)  - College graduate: 11 (16%) | Actual use |
| **O'Brien et al. (2013),** USA | Quantitative  Cross-sectional | DAI (adapted) (29-item)  Likert  Not validated | **n=**18  **Mean age:** 13.90 (SD 3.04) years  **Sex**: Male (9, 50%), female (9, 50%) | Bipolar disorder, depression, ADHD, other | **n=**19  **Mean age:** 43.68 (SD 9.11) years  **Sex**: Male (2, 10.5%), female (17, 89.5%)  **Relationship to child**: all parents  **Education:** NR | Actual use |
| **Post et al. (2002),** USA | Quantitative  Cross-sectional | Author-developed (items NR)  Dichotomous  Not validated | N/A | N/A | **n=**156  **Age:** NR  **Gender**: NR  **Relationship to child:** all parents  **Education:** NR | Hypothetical use |
| **Ricardo Ramírez et al. (2021),** Colombia | Quantitative  Cross-sectional | Author-developed (25-item)  Likert  Not validated | N/A | **Most commonly reported conditions:**  ADHD, intellectual disability, ODD | **n=**98  **Age range**: 19-67 (mean: 39.0, SD 10.49) years  **Gender:** Male (5, 5%), female (93, 95%)  **Relationship to child**: all parents  **Education:**  - Primary: 15 (15.3%)  - Secondary: 50 (51.0%)  - Higher: 33 (33.7%) | Actual use |
| **Stevens et al. (2009),** USA | Quantitative  Cross-sectional | Adapted from Nock and Kazdin (2001) (19-item)  Likert  Not validated | **n=**501  **Age:**  - < 11 years: 232 (46%)  - ≥ 11 years: 269 (54%)  **Gender**: Male (295, 59%), female (205, 41%) | **Primary axis 1 disorders**:  Internalising,  externalising, mixed, other | **n=**501  **Age**: NR  **Gender:** NR  **Relationship to child**: NR  **Education:**  - <11^th^ grade: 218 (43%)  - ≥ High school graduate: 283 (57%) | Hypothetical use |
| **Talbot and Malas (2018),** USA | Quantitative  Pre-post intervention | Adapted from Pescosolido et al. (2007) and Turner (2012) (13-item)  Likert  Not validated | NR | **Most commonly reported conditions:** Anxiety, depression | **n=**30  **Age:**  - 25-34 years: 10%  - 35-44 years: 26.7%  - 45-54 years: 50%  - ≥ 55 years: 13%  **Gender:** Male (10%), female (90%)  **Relationship to child**: all parents  **Education**: all ≥ high school | Hypothetical use |
| **Wallman and Melvin (2022),** Australia | Quantitative  Cross-sectional | Author-developed (6-item)  Likert  Not validated | **n=**143  **Age range**: 12-18 (mean: 15.51, SD 1.74) years  **Gender:** Male (57, 39.9%), female (80, 55.9%), non-binary (6, 4.2%) | NR | **n=**143  **Age range:** 32-58 (mean: 44.25, SD 6.15) years  **Gender:** Male (11, 7.7%), female (132, 92.3%)  **Relationship to child:**  - Biological parent: 138 (96.5%)  - Stepparent: 3 (2.1%)  - Foster-parent/adoptive parent: 1 (0.7%)  - Grandparent: 0 (0%)  - Other: 1 (0.7%)  **Education:**  - Some high school: 7 (4.9%)  - Completed high school: 13 (9.1%)  - TAFE qualification: 43 (30.1%)  - Bachelor’s degree: 31 (21.7%)  - Postgraduate degree: 49 (34.3%) | Hypothetical use |

*ADHD = Attention Deficit Hyperactivity Disorder, ASD=Autism Spectrum Disorder, CBCL = Child Behaviour Checklist, CGI = Clinical Global Improvement, DAI = Drug Attitude Inventory, GAD = Generalised Anxiety Disorder, ICD = International Classification Of Diseases, ITPQ = Initial Treatment Preferences Questionnaire, MDD = Major Depressive Disorder, MH = Mental Health, N/A = Not Applicable NOS = Not Otherwise Specified, NR = Not Reported, OCD = Obsessive Compulsive Disorder, ODD = Oppositional Defiant Disorder, PhD = Doctor of Philosophy, PTSD=Post Traumatic Stress Disorder, TAFE = Technical and Further Education, TPQ-P = Treatment Perceptions Questionnaire – Parent Version, USA = United States of America*

^a^ Mixed methods design, however only quantitative measures relevant to this review

**Appendix 3**

**Table 1***.* Quality appraisal of quantitative descriptive studies (n = 14) using the Mixed Method Appraisal Tool (MMAT) (Hong et al., 2018)

|  | **Q1** | **Q2** | **Q3** | **Q4** | **Q5** |
| --- | --- | --- | --- | --- | --- |
| Al-Haidar (2008) | 🗴 | 🗸 | 🗴 | 🗸 | 🗸 |
| Al-Harthi et al. (2023) | 🗸 | 🗸 | 🗴 | 🗸 | 🗸 |
| Brown et al. (2007) | 🗸 | 🗴 | 🗴 | ? | 🗸 |
| Chandra et al. (2009) | 🗸 | 🗸 | 🗸 | 🗸 | 🗸 |
| Dawood et al. (2020) | 🗸 | 🗸 | 🗸 | ? | 🗸 |
| Jorm and Wright (2007) | 🗸 | 🗸 | 🗴 | 🗴 | 🗸 |
| Langer et al. (2021) | 🗸 | 🗴 | 🗸 | ? | 🗸 |
| Lazaratou et al. (2007) | ? | 🗸 | 🗴 | 🗸 | 🗸 |
| McLaren et al. (2022) | 🗸 | 🗸 | 🗴 | ? | 🗸 |
| O'Brien et al. (2013) | 🗴 | 🗴 | 🗸 | ? | 🗸 |
| Post et al. (2002) | 🗴 | 🗴 | 🗴 | ? | 🗸 |
| Ricardo Ramírez et al. (2021) | 🗸 | 🗸 | 🗴 | ? | 🗸 |
| Stevens et al. (2009) | 🗸 | 🗸 | 🗸 | 🗸 | 🗸 |
| Wallman and Melvin (2022) | 🗸 | 🗸 | 🗴 | ? | 🗸 |
| **Studies meeting each criterion** | 10 | 10 | 5 | 5 | 14 |

Note:

🗸 = Yes

🗴 = No

? = Can’t tell

Critical appraisal questions were as follows:

1. Is the sampling strategy relevant to address the research question?

2. Is the sample representative of the target population?

3. Are the measurements appropriate?

4. Is the risk of nonresponse bias low?

5. Is the statistical analysis appropriate to answer the research question?

**Table 2***.* Quality appraisal of quantitative non-randomised studies (n = 1) using the Mixed Method Appraisal Tool (MMAT) (Hong et al., 2018)

|  | **Q1** | **Q2** | **Q3** | **Q4** | **Q5** |
| --- | --- | --- | --- | --- | --- |
| Talbot and Malas (2018) | 🗸 | 🗸 | 🗴 | ? | 🗸 |
| **Studies meeting each criterion** | 1 | 1 | 0 | 0 | 1 |

Note:

🗸 = Yes

🗴 = No

? = Can’t tell

Critical appraisal questions were as follows:

1. Are the participants representative of the target population?

2. Are measurements appropriate regarding both the outcome and intervention (or exposure)?

3. Are there complete outcome data?

4. Are the confounders accounted for in the design and analysis?

5. During the study period, is the intervention administered (or exposure occurred) as intended?

**Table 3** Quality appraisal of mixed-methods studies (n = 2) using the Mixed Method Appraisal Tool (MMAT) (Hong et al., 2018)

|  | **Q1** | **Q2** | **Q3** | **Q4** | **Q5** |
| --- | --- | --- | --- | --- | --- |
| Hilt et al. (2014) | 🗴 | 🗴 | 🗴 | 🗸 | 🗴 |
| Moses (2011) | 🗴 | 🗴 | 🗴 | 🗸 | 🗴 |
| **Studies meeting each criterion** | 0 | 0 | 0 | 2 | 0 |

Note:

🗸 = Yes

🗴 = No

? = Can’t tell

Critical appraisal questions were as follows:

1. Is there an adequate rationale for using a mixed methods design to address the research question?

2. Are the different components of the study effectively integrated to answer the research question?

3. Are the outputs of the integration of qualitative and quantitative components adequately addressed?

4. Are divergences and inconsistencies between quantitative and qualitative results adequately addressed?

5. Do the different components of the study adhere to the quality criteria of each tradition of the methods involved?
